# How clinical teaching teams deal with educational change: ‘we just do it’

**DOI:** 10.1186/s12909-019-1815-4

**Published:** 2019-10-17

**Authors:** L. Bank, M. Jippes, T. R. van Rossum, C. den Rooyen, A. J. J. A. Scherpbier, F. Scheele

**Affiliations:** 1 Department of healthcare education, OLVG Hospital, Jan Tooropstraat 164, 1061 AE Amsterdam, the Netherlands; 2 Faculty of Science, Athena Institute for Transdisciplinary research, VU University, Amsterdam, the Netherlands; 3 Department of Plastic Surgery, Erasmus Medical Centre, Rotterdam, the Netherlands; 4 Faculty of Health, Medicine and Life Sciences, School of Health Professions Education, Maastricht University, Maastricht, the Netherlands; 5 Movation BV, Maarssen, the Netherlands; 6 School of Medical Sciences, Institute for Education and Training, Amsterdam University Medical Centre, Amsterdam, the Netherlands

**Keywords:** Postgraduate medical education, Clinical teaching teams, Change management, Curriculum change

## Abstract

**Background:**

In postgraduate medical education, program directors are in the lead of educational change within clinical teaching teams. As change is part of a social process, it is important to not only focus on the program director but take their other team members into account. The purpose of this study is to provide an in-depth insight into how clinical teaching teams manage and organize curriculum change processes, and implement curriculum change in daily practice.

**Methods:**

An explorative qualitative semi-structured interview study was conducted between October 2016 and March 2017. A total of six clinical teaching teams (*n* = 6) participated in this study, i.e. one program director, one clinical staff member, and one trainee from each clinical teaching team (*n* = 18). Data were analysed and structured by means of thematic analysis.

**Results:**

The analysis yielded to five factors that positively impact change: shared commitment, reinvention, ownership, supportive structure and open culture. Factors that negatively impact change were: resistance, behaviour change, balance between different tasks, lack of involvement, lack of consensus, and unsafe culture and hierarchy. Overall, no clear change strategy could be recognized.

**Conclusions:**

Insight was gathered in factors facilitating and hindering the implementation of change. It seems particularly important for clinical teaching teams to be able to create a sense of ownership among all team members by making a proposed change valuable for their local context as well as to be capable of working together as a team. Cultural factors seem to be particularly relevant in a team’s ability to accomplish this.

## Background

Whereas many studies examined the impact of educational change on teaching goals and learning outcomes in postgraduate medical education (PGME) [1–3], little is known about the process of how clinical teaching teams (CTTs) implement educational change. In addition to the intended impact of an educational change, it might also affect elements in health care or other areas of daily practice [4, 5]. Insight into how CTT implement change may facilitate future implementation processes in PGME as well as their impact beyond educational boundaries.

Based on the literature and the vast body of research that is being done within several fields [6–10], it can be concluded that change is a multifaceted and challenging process and ways to change more efficiently and successfully are still sought. In a resource-constraint field such as health care, change can be especially challenging. Specifically, educational aspects might be easily overlooked for the sake of clinical expediency in the context of increasing clinical loads [11, 12]. That makes it even more important to make sure educational change happens as efficiently as possible.

It has been shown that change leaders are of great value in implementation processes [7], especially in highly specialized staff groups [13]. However, change is not a one person’s effort, however, rather requires a collective behaviour change in order to benefit the individual, the CTT and possibly even PGME as a whole. Not surprisingly, in several other fields, change is seen as a social process that requires creativity from both employees and management, as well as a sense of employee ownership and support to increase the chances for success [14]. The importance of these elements is also recognized in health care systems with a particular focus on organizational performance and quality improvement [15, 16]. Compared with PGME, change in those settings is far more widespread as the change itself usually involves multiple layers of the organization, affects multiple professions, and the change leaders are mostly actual managers [15, 16].

In PGME, program directors lead CTTs and are responsible for the quality of specialty training within their departments [17–20]. Consequently, they are assigned the responsibility of educational change in those departments as well [17]. Because of their key position, program directors have been the centre of attention in literature on leadership and teamwork [11, 21]. Only a few studies have focused on their role in change processes [22, 23]. However, as change is a social process [14, 21], it is imperative to consider the team as a whole rather than focusing solely on the program director when exploring this subject in PGME. To our knowledge, no studies exist that specifically examined the role of CTTs in implementing educational change. Therefore, the purpose of this study is to provide in-depth insight into how CTTs manage and organize curriculum change processes, and implement curriculum change in daily practice.

## Methods

### Study design

An explorative qualitative interview study was conducted through which data were iteratively collected in semi-structured interviews (see Additional file 1 for the interview guide). More specifically, members of CTTs were interviewed in order to explore their role in implementing educational change. Data were analysed and structured by means of thematic analysis [24].

### Setting

The current study was conducted in the Netherlands. In this country there are eight academic medical centres, each of which coordinates specialty training programs within a geographical region that contains affiliated non-academic teaching hospitals. Postgraduate medical curricula are nationally set and executed by CTTs at local teaching sites. These CTTs consist of a program director, clinical staff members and trainees, and together, the team members are responsible for the quality of teaching and implementing educational change. In general, they have a substantial amount of autonomy in shaping their local training program.

### Participants and sampling procedure

In order to gain insights about change in CTTs, the perspective of all team members was sought, i.e. program directors, clinical staff members and trainees. Previous research has demonstrated that CTTs differ in their change-related behaviour as assessed by the ‘behaviour support-for-change’ measure reflecting the 5 types of change-related behaviour described by Herscovitch and Meyer [17, 25]. The 5 types of behaviour include active resistance, passive resistance, compliance, cooperation, and championing. Team members will commit to a change either because they desire to, have to or ought, which subsequently leads to change-related behaviour. Ideally, this is a type of behaviour that supports the successful implementation of a change initiative, such as compliance, cooperation, or championing. Alternatively, resistance, either passive or active, are forms of change-related behaviour that have a negative impact on the implementation of change. For instance, ‘cooperation’ is defined as demonstrating support by exerting effort into a change, and being prepared to make sacrifices. On the other hand, ‘passive resistance’ is defined as engaging in overt behaviours aimed at preventing the success of a change. CTTs were purposefully selected based on their behavioural support for change score collected during previous research, to increase the sample variety [17]. By selecting CTTs based on this score, and thereby including different types of change-related behaviour, it was expected multiple factors relevant for change processes as well as potential contrasts between CTTs would be recognized.

Program directors received e-mail invitations to discuss participation within their teams. If teams agreed to participate, program directors were asked to put forward both a clinical staff member and a trainee, who were able to provide insight into our research aim. Thereafter, the clinical staff members and trainees received a personal invitation to participate as well. Recruitment of participants continued until data saturation was reached.

### Data collection

All semi-structured single one-on-one interviews were conducted by the main researcher (LB) at private offices at the participants’ workplace between October 2016 and March 2017. The interviews lasted between 25 and 66 min and were audio recorded. Subsequently, they were transcribed verbatim and reviewed for accuracy by the main researcher (LB). Before analysis, all identifying information was removed or anonymized in order to prevent identification of participants in the results presented below.

### Data analysis

The transcripts were coded by attaching keywords, or codes, to all text fragments that were considered relevant for the current study. To allow new insights, open coding was used. The initial interviews focused on the process of initiating change in CTTs and the emerging role of the different team members in relation to change. All interviews were coded by the main researcher (LB). The transcripts of participants from the same CTT were coded consecutively in order to facilitate the recognition of any discrepancies in perceptions or presented facts between the different team members. After the coding of 10 interviews was completed, the derived codes were discussed in the research team, which led to a more in-depth exploration of the program director’s role in the following interviews. More specifically, insights were gathered about the congruence between the current role of the program director and perceptions about what this role should be, and whether that is justly or not.

To enhance reliability, the interviews of one CTT, i.e. the program director, a clinical staff member as well as a trainee (*n* = 3), were analysed by a second researcher (TRvR) using open coding. Differences in coding and selection of text fragments were discussed until consensus was reached. After coding 16 interviews, no new codes were derived. The main researcher (LB) had organised the codes into categories after which the developed coding scheme and derived themes were discussed in depth within the research team until agreement was reached (see Additional file 2 for coding scheme). All coding was performed using qualitative data analysis software (MaxQDA, Version 2007).

### Reflexivity

The first author and main researcher (LB) is a medical doctor and had no personal or professional ties with the participants. All authors are experts in the field of health professions education and well versed in implementation and change management in PGME. Most of them are also actively involved in national committees and organizations responsible for improving and regulating PGME in the Netherlands (AJJAS, CdR and FS).

## Results

### Sample size

A total of nine program directors were asked to participate in our study. Six program directors and their CTTs were willing to participate. From every participating CTT, the program director, one trainee, and one clinical staff member participated, leading to a total of 18 participants. Two program directors, one in pathology and one in radiology, declined participation due to a lack of time and one program director in emergency medicine did not respond to our invitation altogether. Furthermore, two clinical staff members from one CTT refused participation due to a lack of time as well.

The final six participating CTTs (*n* = 6) came from two different non-academic teaching hospitals (*n* = 4), two academic medical centres (*n* = 2), and six different specialties, i.e. Obstetrics and Gynaecology, Internal Medicine, Neurology, Pathology, Radiology and Emergency Medicine. Thereby, Obstetrics and Gynaecology was the only surgical specialty included. Two-thirds of the participants were male, which is in line with the current male/female ratio for practicing medical specialists in the Netherlands [26]. None of the participants had a formal degree in change management. Prior to the interview, participants were instructed to think about the implementation process of a recent educational change that they wanted to discuss during the interview. This request served to stimulate a more in-depth conversation about the *process* of change rather than about the *content* of change. Described changes included the introduction of an electronic portfolio, a new curriculum, and a local quality improvement project. A description of themes that emerged from the data is provided below.

### Change strategies

Generally, all teams recognized that implementing or dealing with change is part of their daily job in both the health care and educational settings. However, despite this awareness, change management support is rarely sought because it is deemed unnecessary or unsuitable. When implementing change, CTTs take a strong pragmatic approach based on trial-and-error rather than on thinking through all possible steps of implementation prior to initiating change. Clinical teaching teams ‘just do it’ with their program director in the lead. That being said, program directors do use elements of change management strategies, albeit without a theoretical background about change. For instance, Kotter’s 8-step change model was the only strategy mentioned and mentioned only once.

### Factors facilitating the implementation of change

When considering all factors that influence the implementation of change in more depth, five facilitating factors were identified (Table 1). When all present, implementing change in a fictional CTT could look like this:

**Table 1:** Factors facilitating the implementation of change

Factors	Quote
Shared commitment	Clinical staff member, interview 9: ‘The intrinsic motivation within our group to provide specialty training is the most important motive for us to implement the necessary changes.’
Reinvention	Program director, interview 3: ‘You try to stay ahead of the crowd. […] You want to help to shape the innovation, I want to be innovative here. […] Otherwise you can only do what others have thought out for you.’
Ownership	Program director, interview 2: ‘When we initiate change, yes, then you need to make enough time available, […]. But above all, you need to have the willingness to make enough time available.’
Supportive structure	Clinical staff member, interview 18: ‘Educational support is crucial I think because you quickly have the tendency to interpret the intended change on your own. […] It is very useful to have the reflection of an educationalist as well.’
Open culture	Trainee, interview 19: ‘I think that you can talk to anybody about anything here. Ideas are always welcome, […], and listened to.’

‘Meet Claire. Claire is an enthusiastic program director. She just found out that she needs to implement Entrustable Professional Activities (EPAs) into her local training program. In order to fully grasp the content and meaning of EPAs she calls in the help of an educationalist. During the next team meeting, she informs her team members, i.e. clinical staff and trainees, about the proposed changes. She takes appropriate time to listen and acknowledges any resistance her colleagues might have. With her enthusiasm, Claire convinces others of the urgency and added value of EPAs, thereby increasing her team members’ willingness to invest time and effort to make this change a success. Both staff and trainees get the opportunity to discuss their ideas and vision. Together, the team determines long-term goals and put a spot on the horizon. A clear change management strategy is lacking; they just do it. In the implementation process that follows, all team members are involved and are delegated a task. Furthermore, a pilot period is introduced in which 2 EPA’s will serve as testcases. By doing so, possible unexpected hurdles will be discovered and dealt with in a timely fashion. From this pilot, they learn and adjust the implementation of the other EPAs in practice. One year later, EPAs are successfully implemented.’

Five main factors that positively impact change were identified. The first was shared commitment; i.e. support, trust, enthusiasm, clear expectations, as well as acknowledgement from the program director are needed to create success. Second, respondents pointed out that it is important to be able to have some influence on how a curriculum change takes shape in their own local context; i.e. to reinvent a change. This also enables them to emphasize their own strengths and to walk ahead of the crowd. In that light, it is important to feel a sense of ownership, which constitutes the third identified factor. People need to have a shared vision, recognize the need for change and put an interest in education. Team members need to feel they are problem owners by talking about the change repeatedly. Additionally, sharing tasks ensures that change is a team effort. Furthermore, in order to change successfully, a clear supportive structure is needed including dedicated time, evaluations, achievable goals. This is also reflected in a change strategy in which the change is repeatedly discussed within the team, and small changes are introduced first in order for team members to get used to a new way of working on a minor scale prior to the implementation of the change in its entirety. Finally, an open culture in which everybody’s opinion is considered valuable positively impacts change processes as well. This also enables a CTT to take a much more flexible approach to change, which includes a clear recognition that things don’t always occur as planned and that adaptation to unexpected occurrences is imperative for obtaining the desired change.

### Factors hindering the implementation of change

Beside the facilitating factors, six discouraging factors were identified as well (Table 2). When all present, implementing change in a fictional CTT could look like this:

‘Meet Fred. Fred is an enthusiastic program director for over 15 years. Just as his fellow program director Claire, he needs to implement EPA’s into his local training program. Fred anticipates is already prepared that this educational change will be met with great reserve and possible even fierce resistance. During the next team meeting, he informs his team members about EPAs, the new nationally set regulations that are to be obeyed. Thereby, he leaves no room for discussion about why or how EPAs are implemented. Clinical staff members show discontent but are mainly concerned about whether this change will interfere with their daily work. His trainees remained largely silent as they are the direct object after all. A vision, a timescale and long-term goals are not set. Not surprisingly, in the implementation process that follows, nobody feels responsible and teamwork is lacking. One year later, EPAs are nowhere near implemented.’

**Table 2:** Factors hindering the implementation of change

Factors	Quote
Resistance	Program director, interview 16: ‘The most important changes, you just push them trough. And then starts the pushing and shoving, the sabotaging. […] Just see how much you can win back from what you had before, that is what is going on.’
Disbalance in tasks	Trainee, interview 13: ‘They (clinical staff) know about CBME, but there is a difference between knowing and doing. If my individual training program interferes with their clinical practice, my training is sacrificed for their logistics.’
Behavior change	Program director, interview 6: ‘The translation of a change into actual behavior change, I experience myself how much effort that costs. That I know I need to do things, but that it is not internalized yet.’
Lack of involvement	Clinical staff member, interview 11: ‘I first heard of EPAs on a symposium. After that, I asked the program director about EPAs. He said that he wanted to implement them shortly. I thought, why don’t I know about this? Just tell us that. During the symposium I also found out that the preparations for EPAs were almost ready and they would be implemented within 2 weeks. Really weird I didn’t know this.’
Lack of consensus	Program director, interview 10: ‘I have the feeling that it (the implementation of CBME) is very much laid down on us from top down. Without people asking us, are we alright with this.’
Unsafe culture and hierarchy	Trainee, interview 13: ‘You are enormously dependent on them (clinical staff). They say, you need to swallow anything for 5 years. If you do that, you will have a nice career, if not […], you will feel that for the rest of career. For instance because they give you bad references. That is a sort of hidden rule.’

Six main factors that negatively impact change were identified. The first was resistance. Described resistance behaviours ranged from the initial grumbling and sighing to clear refusal and active sabotaging change efforts. Interestingly, the initial resistance seemed to be less intense when a change was the result of, for instance, national regulations, rather than the initiative of a local team member. In the latter case, team members felt more room to negotiate change conditions. Second, respondents pointed out that changing one’s own behaviour is really difficult as it requires adjustment of personal routines. It is something one consciously needs to work on and takes dedicated time and effort. In that light, the balance between all different tasks CTTs have, the third factor identified, could make change even harder. Time constraints and demands in patient care could subvert change efforts and make team members easily slip back into old habits. Fourth, the feeling of not being involved made respondents less willing to accept a change, reduced the feeling of ownership, and clearly undermined teamwork. Furthermore, a lack of consensus and the feeling that a change is just introduced without one’s approval gave respondents the feeling that they were not in control over an unreasonable change. Again, this undermined teamwork as well. Finally, an unsafe culture and hierarchy impacted change processes quite vigorously. Hierarchy between trainees and staff as well as the dependency of trainees on their supervisors resulted in trainees refraining from voicing their opinions out of fear for a negative impact on their assessment or even their future career. The influence of hierarchy between staff members was also mentioned to have a negative impact on change, where mainly younger clinical staff members were more easily influenced by their older colleagues.

### Role patterns

When looking at the different roles within CTT, the program director clearly has the lead in change processes and is the first to receive information about a change. Furthermore, the program director is responsible for transforming new regulations into practical and reachable changes in their own local context. Clinical staff members fully depend on the information provided to them by the program director as they don’t have and don’t look for access to the right sources of information. This is mainly due to a strong division of tasks between staff members, where the program director is appointed to be in charge of training matters and is to receive full support from his/her team members to perform this task. Generally, clinical staff members tend to just comply with a change as long as it does not interfere with their daily work. On the other hand, the extent to which trainees can take an active role in change implementation depends to a large extent on the responsibilities given to them by the program director. As mentioned above this is also closely related to culture within the CTT.

### Program director as manager

During the interviews, it became clear that program directors accept the role of program director mainly from an idealistic point of view in which they want to contribute to the education of trainees by actually educating and teaching them on a daily basis. In reality, however, this role is more that of a manager who ensures that the circumstances to facilitate PGME are as optimal as possible. This means, for instance, paying attention to and fostering a safe learning climate, motivating clinical staff members, and keeping track of the overall progression of all individual trainees. Even though program directors acknowledge these tasks are currently part of their job description, they do add the footnote that this does not mean that they are automatically competent to perform these tasks as they were not trained as ‘manager’. It is just assumed program directors can.

## Discussion

An explorative study about how CTTs in PGME deal with curriculum change in daily practice was performed. By taking a closer look at the different team members, insights were obtained in their roles, as well as in cultural and other factors that influence the implementation of change, and the strategies applied.

Overall it can be concluded that no clear change strategy appears to be utilized. Previous research has already shown that the practice of including change management in change processes in PGME is rather limited [17, 27]. Program directors, who are in the lead of these change processes [17, 22, 27], do use elements of change management strategies, however, showed limited awareness of their own approaches regarding these strategies. This raises the question whether program directors are able to adequately adapt their actions to the circumstances, since awareness of the effect on one’s actions is a required first step to subsequent behavioural change. Being able to adapt a change strategy is considered important for a successful implementation process, as it will enable program directors to tailor the implementation process to the needs of those involved, to address discrepancies between them, and thereby to increase a sense of ownership [7, 9, 10]. Indeed, it has been suggested before that program directors might be rigid in adapting their approaches to change to specific circumstances [22]. On the other hand, our results also revealed that an open culture within CTTs seems to facilitate a flexible approach to change and the ability to adapt.

The factors influencing change processes that were identified in this study were comparable to factors that have been described previously in other health care and educational settings [7, 12, 17, 28, 29]. Therefore, the factors are not to be perceived as new, but rather viewed as important in the context of implementing change in PGME. When looking at the facilitating factors in more depth, it can be concluded that these factors enable CTT to exert high quality teamwork and make a proposed change valuable for their local context by creating strong ownership within the team [7, 14]. Cohesive teamwork leads to higher levels of knowledge sharing, enthusiasm, and it facilitates the development of shared meanings and values in relation to the change, all of which increases productivity and the ability to change [7, 12, 19]. Indeed, the relevance of teamwork has also been recognized in changing quality improvement systems [16]. Furthermore, teams with a shared purpose and who create opportunities for participation and input from all team members are much more likely to create collectivity within their teams [21]. In order to do so, the team will need to be able to learn collaboratively, i.e. learning from mistakes and being able to evaluate and modify the initial plans [7, 21]. However, it has been shown that collaborative learning is not something that comes naturally to program directors, as making mistakes and showing vulnerability does not fit the image of an autonomous professional. Furthermore, high quality teamwork also requires some individual autonomy to be sacrificed for the greater good of the team’s mission and goals [11]. As medical doctors highly value autonomy, this can also be very challenging, especially in case of an unsafe culture involving a strong hierarchy [11, 18, 19, 30]. In fact, strong hierarchies, as shown in our results, can even lead to the exclusion or suppression of colleagues, particularly trainees. Indeed, previous research has shown that there is a positive relationship between a receptive culture and innovativeness in multiple contexts [31–33]. Not surprisingly, literature suggests that implementation outcomes, organizational effectiveness and receptiveness to change are affected by organizational culture [28, 34–36]. Despite the fact that it is not possible to describe the relative importance of the factors in relation to each other, a potential cumulative effect cannot be excluded. During the interviews, all participants were able to identify multiple factors either facilitating or hindering implementation processes suggesting establishing change is a balance between multiple factors. Moreover, cultural aspects in particular seemed to either facilitate or hinder teamwork more generally. Indeed, previous research has shown change processes represent a delicate interplay between influencing factors in a complex setting, such as attributes of the innovation itself and characteristics of the organization [7, 12, 28].

The medical habitus, i.e. the common professional identity or the way doctors behave according to the specific norms of the profession [18, 37], might also influence the program directors’ ability to lead change. This informal medical culture comes with some collegial manners, which include not to give orders, not to control each other, to make consensual decisions, to be loyal to each other, and not to criticize each other openly. This common habitus could potentially be the breeding ground for competition. By not following the appropriate collegial strategies, medical doctors risk conflicts and the performance of the group [18, 30]. During a change process, a program director might not be able to follow all collegial manners and needs to be able to manage conflict in a way that stimulates collaborative learning [21] instead of undermining it and potentially derail the implementation process [11]. Training in communication, conflict management, as well as in building collectivity might be helpful for program directors and their fellow team members to perform high quality teamwork and subsequently facilitate change implementation [7, 11, 21, 30]. Besides, faculty development should also focus on change management principles to further equip faculty to implement change. For trainees, on the other hand, leading change is already one of the key concepts of the competency framework CanMEDS [38]. However, this role mainly refers to contributing to the improvement of health care delivery and cost-efficiency. Enabling competencies described include ‘analyze patient safety incidents’ or ‘apply the science of quality improvement’. The ability to use insights from change management as such is not mentioned in these competencies [38]. From our perspective, the value of knowledge and skills rooted change management principles should be acknowledged and be more specially integrated in training programs.

At last, in line with the literature, we found that external pressures to change increase an organization’s motivation to change but not its capacity [7]. In our results this is reflected in the fact that changes related to national regulations not only reduce the level of initial resistance, however, also diminish the feeling of involvement and consensus, two factors that negatively influence change processes.

### Strength and limitations

To our knowledge, this is the first study to explore the approaches to change of the CTT as a whole. As change requires high quality teamwork, it is important to not solely focus on the program director. Another strength of this study was that the interviews were conducted with all different team members separately in order for them to speak freely about their thoughts and experiences. The results of this study showed that an unsafe culture and hierarchy influenced trainees in their ability to express their opinions. Therefore, interviews with all team members together could have hindered an open discussion. Furthermore, this interview method was meant to limit the potential effect of the recruitment method on the results. In this study, program directors were asked to put forward other participants. Thereby, program directors could potentially influence the results, for instance, by suggesting colleagues who would speak more positively about current change processes.

As in all qualitative studies, this study is limited in its generalizability. As the organization of PGME might differ between countries, not all results might be relevant in other settings. However, as curriculum change is a continuous factor in PGME all around the world, it is our belief that the gained knowledge could be of importance in planning change and implementation in other settings as well.

Due to our method of data collection, the results potentially present a limited picture of change in CTTs. In interviews, participants describe their anticipated response, which may differ from their actual response when encountering a similar situation. Empirical work could be strengthened by observational studies to further analyse the use of change strategies in practice. Additionally, action research could provide valuable insight into what kind of change strategy helps to improve implementation efforts in CTTs.

## Conclusions

In this semi-structured interview study, an insight was gathered in factors facilitating and hindering the implementation of change. Program directors showed limited awareness in the change strategies they apply. It is unclear whether they are able to adapt their strategies appropriately when needed, which potentially harms the efficiency of the implementation process. On the other hand, results suggest that it is particularly crucial to create a sense of ownership among all team members as well as be able to exert teamwork by learning collectivity. Cultural factors seem to be particularly relevant in a CTTs ability to accomplish this.

## Supplementary information

**Additional file 1.** Interview guide.

**Additional file 2.** Coding scheme.

## Data Availability

The datasets used and analysed during the current study are available from the corresponding author on reasonable request.

## Abbreviations

CTT: Clinical teaching team. Within a teaching hospital, the different medical specialties provide specialty training within their own departments. The medical doctors in these departments together form a clinical teaching team and are responsible for providing these up to standard specialty training programs. A clinical teaching team consists out of a program director, all clinical staff members and all trainees within that department.
PGME: Postgraduate medical education
