# Supplementary material for: How clinical teaching teams deal with educational change: ‘we just do it’
Source: BMC Med Educ. 2019 Oct 17;19:377. doi: 10.1186/s12909-019-1815-4 (PMC6796387; doi:10.1186/s12909-019-1815-4)
Supplement: Supplementary file 1 — Additional file 1. Interview guide. [file 12909_2019_1815_MOESM1_ESM.docx]

**Additional file 1 – Interviewguide**

Part 1 – Change process in postgraduate medical education

*1) Topic: recent change in the local setting*

- As preparation for this interview I asked you to think about a recent curriculum change you went through and the factors, situations or else that influenced the change process. First can you tell me what came to your mind?
- Why were these factors, situations or else important?
- What was their influence on the change process?

*2) Topic: different roles within the clinical teaching team*

- What is the influence of the different clinical teaching team members, i.e. program director, clinical staff and trainees, on a curriculum change?
- Do you recognize different role patterns?
- Does the role of the program director differ from the other team members?
- How do you see your own role within the team?
- If different role patterns are described: where do you think these differences come from?
- Do you experience any resistance to curriculum change within the team? How is this expressed by you and/or others?
- What is the main reason resistance to change does or doesn’t arise?

*3) Topic: what is needed to change in the local setting*

- Do you experience there are enough opportunities to change? Can you explain that?
- What do you think you need in order to successfully implement curriculum change? Did you miss any of these things in the recent change you went through? Can you explain that?

Part 2 – External pressure

*1) Topic: relevance of external pressure*

- In previous research we found that external pressure, i.e. pressures from outside the clinical teaching team such as hospital boards and the ministry of Education, are not considered relevant for change in postgraduate medical education. Can you help us to explain this result? What are you views on this topic? What is your own experience in regards to external pressures in practice?
- It also became clear that the program director is in the lead when is comes to curriculum change processes. Do you recognize this and can you support you answer with an example?

Part 3 – The role of the program director

*1) Topic: expectations and job description*

For program directors:

- When you became a program director, how did you think your job would look like? Does that match the reality?
- Over the years, the role of the program director has become more and more that of a manager. Do you feel you are competent to fur fill these tasks? Can you explain that?
- Do you think the management tasks program directors have nowadays, should be part of their job description and can you tell me why or why not?

For the other team members:

- Over the years, the role of the program director has become more and more that of a manager. Do you think program directors are competent to fur fill these tasks? Can you explain that?
- Do you think the management tasks program directors have nowadays, should be part of their job description and can you tell me why or why not?
