# Supplementary material for: How clinical teaching teams deal with educational change: ‘we just do it’
Source: BMC Med Educ. 2019 Oct 17;19:377. doi: 10.1186/s12909-019-1815-4 (PMC6796387; doi:10.1186/s12909-019-1815-4)
Supplement: Supplementary file 2 — Additional file 2. Coding scheme. [file 12909_2019_1815_MOESM2_ESM.docx]

**Additional file 2 – Themes, categories and sub-categories used in the analysis**

| **Theme** | **Category** | **Sub-category** |
| --- | --- | --- |
| Context | Change is everywhere |  |
|  | Organizational consequences of change |  |
| Program director as manager | Expectations |  |
|  | Capabilities |  |
|  | Part of task | Yes  No |
| Action plan | Just do it |  |
|  | Activities | Create shared responsibility   - Create social pressure - Involvement of trainees - Exert pressure - Stress importance - Addressing the issue   Create structure   - Adjusting - Discuss regularly |
| Factors influencing the implementation of change | Facilitating | Shared commitment   - Enthusiasm - Cooperation / support - Acknowledgement - Dedicated management - Clear expectations - Discuss in a timely fashion   Reinvention   - Walk ahead of the crowd - Room for your own interpretation   Ownership   - Need for change - Shared vision - Shared goals - Shared responsibility - Importance of teaching - Clear wins - Trainees’ familiarity with tools   Supportive structure   - Reasonable - Dedicated time - Structural part of working procedure - Evaluation - Clear relevance - Good facilities in hospital   Open culture |
|  | Hindering | Resistance   - Obstinacy - Emotions - Bullying / make trouble - Tensions related to other topics - Dependency / hierarchy - Insufficient compliance   Disbalance in tasks   - Teaching has no priority - Patientcare versus training - Resources: time, money, training   Behaviour change   - Relapse into old routines - Major adjustments of routines - Loss of structure   Lack of involvement   - Not informed - No leadership   Lack of consensus   - Change does not match one’s own vision - No support from program director - Relevance not recognized - No consensus - Depending on only a few people - Lack of transparency   Unsafe culture and hierarchy |
| Role patterns | Program director | Information   - Information source - Come up with initiatives   Support   - Motivation - Offer tools - Lead by example - Reassuring - Controlling - Insisting - Control versus let go - Find meaning - Reinforce leadership role |
|  | Trainee | Active   - Contributing - Protest - Controlling   Passive   - Subject - Length of training period - Patient care - Number of years in training |
|  | Faculty | Active   - Participating - Every faculty member is a teacher   Passive   - Subject |
| Information channels | Informal | Best practices  Backrooms / rumours  Equals |
|  | Formal | Scheduled meetings  Mediation  Training  National or regional forums  Digital |
| External influences | Lack of incentive |  |
|  | Not necessary |  |
|  | Unknown |  |
